# Non-Coding RNAs Operate in the Crosstalk Between Cancer Metabolic Reprogramming and Metastasis

**DOI:** 10.3389/fonc.2020.00810

**Published:** 2020-05-29

**Authors:** Ziyi Li, Xueying Sun

**Affiliations:** ^1^The Hepatosplenic Surgery Center, The First Affiliated Hospital of Harbin Medical University, Harbin, China; ^2^Department of Molecular Medicine and Pathology, Faculty of Medical and Health Sciences, The University of Auckland, Auckland, New Zealand

**Keywords:** non-coding RNA, metabolic reprogramming, cancer metastasis, microRNA, long non-coding RNA

## Abstract

Metastasis, the spread of cancer cells from a primary tumor to a secondary site, represents one of the hallmarks of malignancies and the leading cause of cancer-related death. The process of metastasis is a result of the interaction of genetic heterogeneity, abnormal metabolism, and tumor microenvironments. On the other hand, metabolic reprogramming, another malignancy hallmark, refers to the ability of cancer cells to alter metabolic and nutrient acquisition modes in order to support the energy demands for accomplishing the rapid growth, dissemination, and colonization. Cancer cells remodel metabolic patterns to supplement nutrients for their metastasis and also undergo metabolic adjustments at different stages of metastasis. Genes and signaling pathways involved in tumor metabolic reprogramming crosstalk with those participating in metastasis. Non-coding RNAs are a group of RNA molecules that do not code proteins but have pivotal biological functions. Some of microRNAs and lncRNAs, which are the two most extensively studied non-coding RNAs, have been identified to participate in regulating metabolic remodeling of glucose, lipid, glutamine, oxidative phosphorylation, and mitochondrial respiration, as well as the process of metastasis involving cell motility, transit in the circulation and growth at a new site. This article reviews recent progress on non-coding RNAs operating in the crosstalk between tumor metabolic reprogramming and metastasis, particularly those influencing metastasis through regulating metabolism, and the underlying mechanisms of how they exert their regulatory functions.

## Introduction

Metastasis is one of the important cancer hallmarks (1), and a complex multistep process involving intracellular and intercellular signal transduction cascades and comprising the proliferation of primary tumor cells, endovascular intervention, the formation of pre-metastatic niches, and subsequent dissemination of cancer cells or micro-metastases into distant organs (2, 3). Metastasis contributes largely to the mortality for many major cancer types, and exploring the underlying mechanisms is of great significance for seeking effective treatments and improving the prognosis of cancer patients.

**Figure 1:**
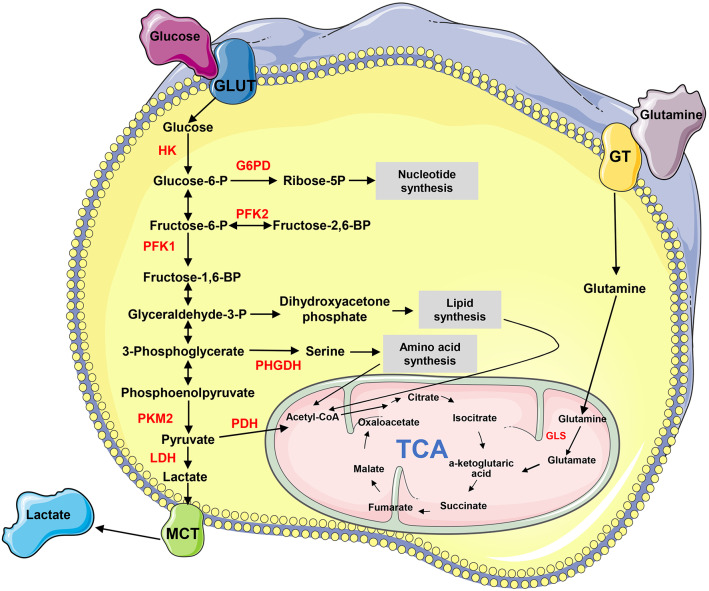
The biochemistry of metabolism in cancer cells. “→” indicates positive regulation or activation. Key enzymes in energy metabolism are marked as red color. G6PD, glucose 6-phosphate dehydrogenase; GLS, glutaminase; GLUT, glucose transporter; GT, glutamine transporter; HK, hexokinase; LDH, lactate dehydrogenase; MCT, Monocarboxylate transporter; PDH, pyruvate dehydrogenase; PFK, phosphofructokinase; PHGHD, D-3-phosphoglycerate dehydrogenase; PKM2, pyruvate kinase M2; TCA, tricarboxylic acid cycle.

Metabolism transforms the absorbed nutrients into small molecule metabolites to sustain the stability of homeostasis, generate bioenergy and regulate cell signaling pathways and physiological activities (4). Metabolic reprogramming is a malignancy hallmark, which refers to the ability of cancer cells to adjust metabolic and nutrient acquisition modes to support their rapid growth, dissemination, and other characteristics (5). The metabolites, such as glucose, amino acids, nucleic acids, and lipids, are remodeled in tumors (Figure 1). Cancer metastasis usually accompanies metabolic reprogramming, and this metabolic change has been recognized at different stages of metastasis (6, 7). Non-coding RNAs (ncRNAs), particularly long-chain non-coding RNA (lncRNA) and microRNA (miRNA), play important roles in regulating metabolic remodeling and the metastasis of cancer cells (8–11). This article aims to discuss the recent progress of how lncRNAs and miRNAs orchestrate in the crosstalk between cancer metabolic reprogramming and metastasis, particularly those influencing metastasis through regulating metabolism-related genes and signaling pathways.

## Metabolic Reprogramming and Cancer Metastasis

In the 1920s, Warburg observed a special phenomenon that even in aerobic conditions, cancer cells tend to favor metabolism relying on glycolysis rather than the much more efficient oxidative phosphorylation pathway, which is the preference of most normal cells (12, 13). As a result, tumor cells have significantly increased glucose uptake and secretion of lactate, which is converted from pyruvate, the last product of glycolysis (14). This process, now known as the Warburg effect or aerobic glycolysis, marks the start of a new era for studying tumorigenesis and cancer progression in terms of metabolism. Gene mutations, deletions, and translocations have an impact on various signal pathways in cancer cells, and the main oncogenic signal pathways will finally converge to metabolism (15, 16). Metabolites by cancer cells not only supply materials for their proliferation and metastasis but also provide sustaining signals to meet their survival needs in tumor-specific microenvironments (1, 17, 18). In addition, cancer cells affect the metabolism of distant organs, where they metastasize, to facilitate their implantation and growth (19). On the other hand, metastatic cancer cells are also affected by the metabolic alterations of invaded organs and tissues (20, 21).

Tumor cells are characterized by high aerobic glycolysis and high oxidative stress (16). Studies have found that the Warburg effect inhibits anoikis and promotes tumor metastasis (22, 23). Meanwhile, aerobic glycolysis increases glucose consumption, reduces the generation of excessive reactive oxygen species (ROS), and enhances the antioxidant capacity of tumor cells, which obtain the ability to resist anoikis and metastasize (22). Despite the Warburg effect, oxidative metabolism is also a major source of adenosine triphosphate (ATP) in some tumors (24, 25). Mitochondrial oxidative metabolism produces ROS including the hydroxyl (HO^−^) free radicals, superoxide (${\text{O}}_{2}^{-}$) and non-radical molecules such as hydrogen peroxide (H_2_O_2_) (26). Low or moderate levels of ROS in tumor cells can activate a series of signaling pathways, causing genomic DNA mutations and promoting tumor formation and progression; but high levels of ROS can lead to the death of neoplasm cells (27). The roles of ROS in tumor metastasis remain controversial among different studies. One study shows that antioxidants can promote tumor metastasis (28). Melanoma metastatic cells express higher levels of lactic acid transporter and absorb more lactic acid, enabling them to produce more antioxidants and survive in the blood (29). On the other hand, some studies demonstrate that antioxidants inhibit tumor metastasis, suggesting that ROS may promote the dissemination of tumor cells (30). H_2_O_2_ has also been shown to promote tumor metastasis by inhibiting anoikis of detached cancer cells (31).

## Roles of ncRNAs in Cancer

**Figure 2:**
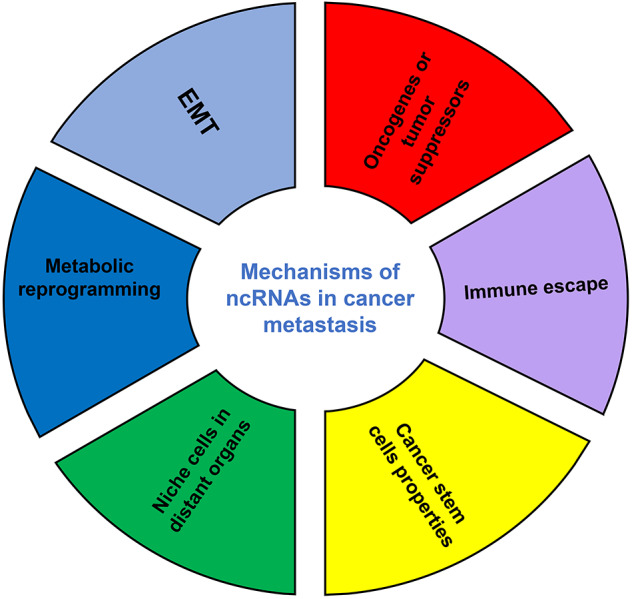
The mechanisms of ncRNAs involved in cancer metastasis. EMT, epithelial-mesenchymal transition.

Although the number of ncRNAs in the human genome is unknown, recent studies suggest that thousands of ncRNAs exist in the body. At present, a dozen of abundant and functionally important types of ncRNAs have been discovered, among which miRNA and lncRNA are the two most extensively studied. Because they do not encode proteins, ncRNAs were initially considered to lack biological functions. However, it's now clear and well accepted that ncRNAs affect the development and progression of many malignancies by regulating the proliferation, apoptosis, differentiation, and metastasis of cancer cells (32) (Figure 2). Accumulating evidence also indicates a link between ncRNAs and metabolic changes in cancer (33, 34) (Table 1). NcRNAs are shown to regulate key metabolic enzymes and signaling pathways involved in metabolic reprogramming, resulting in tumorigenesis, cancer progression and metastasis (60, 61).

**Table 1:** NcRNAs involved in regulating tumor metabolism.

	**NcRNAs**	**Targets**	**Actions**	**Tumor types**	**References**
**Glucose metabolism**					
MiRNAs	miR-199a-5p	HK2	Down	HCC	(35)
	miR-139-5p	ETS1	Down	HCC	(11)
	miR-122	PK, GLUT1	Up	Breast cancer	(19)
	miR-122	Aldolase A	Down	HCC	(36)
	miR-125b	HK2, et al.	Down	CLL	(37)
	miR-30a-5p	LDH	Down	Breast cancer	(38)
	miR-483, miR-551a	CKB	Down	CC	(21)
	miR-361-5p	FGFR1	Down	Breast cancer	(39)
LncRNAs	TUG1	miR-455-3p	Up	HCC	(40)
	PVT1	miR-143	Up	Gallbladder cancer	(41)
	FEZF1-AS1	PKM2	Up	CRC	(42)
	lnc-p23154	miR-378a-3p	Up	OSCC	(43)
	lnc-IGFBP4-1	LDH	Up	LC	(44)
	SAMMSON	P32	Interact	Melanoma	(45)
	MALAT1	TCF7L2	Up	HCC	(46)
	LINC00092	PFKFB2	Up	OC	(47)
**Lipids metabolism**					
MiRNAs	miR-18a-5p	SREBP1	Down	Breast cancer	(48)
	miR-661	StarD10, Nectin-1	Up	Breast cancer	(49)
	miR-195	ACC, FASN	Down	Breast cancer	(50)
	miR-409-3p	FABP4	Down	OC	(51)
	miR-22	ACLY	Down	Breast cancer	(52)
	miR-133b	PPARγ	Down	Gastric cancer	(53)
LncRNAs	HULC	miR-9	Up	HCC	(54)
**Glutamine metabolism**					
MiRNAs	miR-181d	CRY2, FBXL3	Up	CRC	(55)
	miR-23b	Proline oxidase	Up	Prostate cancer	(56)
LncRNAs	GLS-AS	GLS	Down	PC	(10)
	OIP5-AS1	miR-217	Up	Melanoma	(57)
	XLOC_006390	c-Myc	Up	PC	(58)
	UCA1	miR-16	UP	Bladder cancer	(59)

*Up/Down indicates that ncRNAs upregulate or downregulate the expression of target genes. ACC, acetyl-CoA carboxylase; ACLY, adenosine triphosphate citrate lyase; CC, colon cancer; CKB, creatine kinase, brain-type; CLL, chronic lymphocytic leukemia; CRC, colorectal cancer; CRY2, cryptochrome circadian regulator 2; ETS1, E26 transformation-specific 1; FABP4, fatty acid binding protein 4; FASN, fatty acid synthase; FBXL3, F-box and leucine rich repeat protein 3; FGFR1, fibroblast growth factor receptor; GLS, Glutaminase; GLUT1, glucose transporter 1; HCC, hepatocellular carcinoma; HK2, hexokinase 2; LC, lung cancer; LDH, lactate dehydrogenase; OC, ovarian cancer; OSCC, oral squamous cell carcinoma; PC, pancreatic cancer; PFKFB2, 6-phosphofructo-2-kinase/fructose-2,6-biphosphatase 2; PK, pyruvate kinase; PKM2: pyruvate kinase isozymes M2; PPARγ, peroxisome proliferator-activated receptor-γ; SREBP1, sterol regulatory element binding transcription protein 1; TCF7L2, transcription factor 7-like 2*.

## ncRNAs in Metabolic Reprogramming and Cancer Metastasis

### Glucose Metabolism

Cancer cells have significantly enhanced glycolysis during the metastatic process and the glucose supply is dramatically reduced in solid tumors (12, 13). The intermediate metabolites generated in glycolysis are important synthetic materials for tumor growth (62). The acidic and hypoxic microenvironment (TME) inside solid tumors promotes invasion and immune escape (63, 64) by regulating the function and subcellular localization of cytoskeleton proteins, thus promotes the invasion and metastasis of tumor cells through protonation of critical pH-sensitive residues (65, 66). In addition, aerobic glycolysis reduces the oxidative metabolism of glucose, making cancer cells resistant to anoikis and promoting the survival of circulating tumor cells (67).

The first step in glycolysis is the phosphorylation of glucose to form glucose 6-phosphate by a family of enzymes called hexokinases (HKs), which are key glycolytic enzymes to control the rate of glucose metabolism and highly expressed HKs maintain a speedy glycolytic rate in tumor tissues, helping the metastasis of cancer cells (68). HK2 can regulate the expression of matrix metalloproteinase (MMP)-9, SRY-box transcription factor (SOX)-9 and non-processed pseudogene (NANOG) and facilitate the metastasis of ovarian cancer cells (69). Lower expression of miR-139-5p correlates with a worse prognosis of hepatocellular carcinoma (HCC) and overexpressed miR-139-5p restrains aerobic glycolysis, suppressing the metastasis of HCC cells (11). In a mechanism exploration, miR-139-5p regulates the expression of HK1 and 6-phosphofructo-2-kinase/fructose-2,6-biphosphatase (PFKFB) 3 by directly targeting ETS proto-oncogene 1 (ETS1), a transcription factor binding to the promoters of HK1 and PFKFB3 genes, while ETS1 silencing induces the expression of miR-139-5p via a post-transcriptional regulation mode involving Drosha (11). Knockdown of lncRNA-TUG1 induces a marked inhibition of cell migration, invasion, and glycolysis by suppressing miR-455-3p, which is transcriptionally repressed by p21 and directly targets the 3′-UTR of adenosine monophosphate-activated protein kinase subunit β2 (AMPKβ2), thus the lncRNA TUG1/miR-455-3p/AMPKβ2 axis regulates the metastasis and glycolysis of HCC cells through the regulation of HK2 (40). LncRNA PVT1 is upregulated in gallbladder cancer (GBC) tissues and negatively associated with the overall survival of patients. Knockdown of lncRNA PVT1 inhibits the metastasis of GBC cells by regulating aerobic glucose metabolism via modulating HK2 expression by competitively binding to endogenous miR-143 as a competing endogenous RNA (ceRNA) (41).

Pyruvate kinase M2 (PKM2) is an alternatively spliced variant of pyruvate kinase that is preferentially expressed during embryonic development and in many types of cancer cells. PKM2 alters the final rate-limiting step of glycolysis, resulting in a cancer-specific Warburg effect, thus determining the efficiency of glucose utilization and the production of lactic acid (70). The direct interaction of PKM2 in the nucleus with TGF (transforming growth factor)-β-induced factor homeobox 2 (TGIF2), leading to the recruitment of histone deacetylase 3 to the E-cadherin promoter sequence, with subsequent deacetylation of histone H3 and suppression of E-cadherin transcription, thus promoting the process of epithelial-mesenchymal transition (EMT) in colon cancer cells (71). MiR-let-7a inhibits the migration of cervical cancer cells through downregulating PKM2 (72). Breast cancer cells secret miR-122-carrying vesicles into circulation to facilitate their metastasis by increasing nutrient availability in pre-metastatic niches in distant organs including brains and lungs. Mechanistically, the cancer-cell-derived miR-122 suppresses glucose uptake by niche cells via downregulating the glycolytic enzyme pyruvate kinase (19). LncRNA FEZF1-AS1 is one of the most highly expressed lncRNAs in colorectal cancer (CRC) and exerts a promoting function on the metastasis of CRC cells. FEZF1-AS1 binds to and stabilizes the PKM2 protein, thereby activating the STAT3 (signal transducer and activator of transcription 3) signaling pathway and increasing aerobic glycolysis (42).

Glucose transporter (GLUT) is a transmembrane glycoprotein distributed on the cell membrane and mainly carry out transmembrane transport of glucose, thus represents a key factor for cancer cells to take up glucose. High expression of GLUT1 promotes the transport and absorption of glucose, providing abundant materials for glycolysis, thereby enhancing the metastasis of cancer cells (73). GLUT1 gene expression is associated with the invasiveness and MMP-2 activity in pancreatic cancer (74). MiRNAs mediate fine-tuning of genes including GLUTs involved in cancer metabolism to support the biosynthetic and energy requirements for the metastasis of cancer cells (75). MiR-122 secreted by breast cancer cells restricts the expression of GLUT1 in non-cancerous brain astrocytes and lung fibroblasts, allowing cancer cells to obtain sufficient glucose supply during the processes of lung and brain metastases (19). LncRNA lnc-p23154 is shown to be associated with the metastasis of oral squamous cell carcinoma (OSCC) and promotes OSCC cell migration and invasion by suppressing the expression of GLUT1 via its negative effects on miR-378a-3p, which has an inhibitory effect on the expression of GLUT1 (43).

Lactate dehydrogenase A (LDHA) is another important rate-limiting enzyme in glucose metabolism by catalyzing the interconversion of pyruvate and lactate. The phosphorylation of LDHA at Y10 is positively correlated with the progression of metastatic breast cancer (76). LDHA can promote the process of EMT by activating EMT-related proteins and facilitate the metastasis of lung adenocarcinoma (77). In breast cancer cells, miR-30a-5p suppresses LDHA expression by directly targeting its 3′-UTR, thus inhibits glycolysis by decreasing glucose uptake, lactate production, ATP generation, and extracellular acidification rate, and increasing oxygen consumption. As a result, glycolysis regulated by miR-30a-5p plays a critical role in the metastasis of breast cancer cells (38). Lnc-IGFBP4-1 promotes the metastasis of lung cancer cells through a possible mechanism of metabolic reprogramming by enhancing the expression of LDH and ATP production (44).

In addition to the above glycolytic enzymes, other kinases in glycolysis are also regulated by ncRNAs. The high level of glucose uptake and aerobic glycolysis stimulates the hexosamine biosynthetic flux, leading to an increased level of UDP-β-DN-acetylglucosamine (UDP-GlcNAc), which is the final product of hexosamine biosynthesis (78, 79) and catalyzed by O-GlcNAcylation transferase (OGT) (80). OGT is post-transcriptionally inhibited by miRNA-101 and upregulated OGT increases O-GlcNAcylation level and promotes the metastasis of CRC cells (81). MiR-551a and miR-483 suppress hepatic colonization and metastasis of CRC cells by convergently dysregulating creatine kinase brain-type (CKB), which is released into the extracellular microenvironments by hypoxic metastatic cells and catalyzes the production of phosphocreatine that helps to generate ATP and fuel metastatic cells (21). In ovarian cancer, the chemokine CXCL14 (C-X-C motif ligand 14)-high expressed cancer-associated fibroblasts mediate the upregulation of lncRNA LINC00092, which downregulates PFKFB2 (6-phosphofructo-2-kinase/fructose-2,6-biphosphatase 2), a glycolytic enzyme involved in synthesis and degradation of fructose-2,6-bisphosphate, thereby promoting metastasis by altering glycolysis (47). MiR-361-5p inhibits the glycolysis and invasion of breast cancer cells by respectively targeting MMP-1 and fibroblast growth factor receptor 1 (FGFR1), which is a promoter of glycolytic enzyme and a suppresser of OXPHOS (oxidative phosphorylation) (39).

### Mitochondrial Respiration and Oxidative Phosphorylation

Tumor cells employ glycolysis as the main energy supply method even in oxygen-rich environments due to their impaired mitochondrial function, along with the reductive carboxylation of glutamine to malate (12). Hypoxia and reduced mitochondrial capacity promote cancer cell dependence on glycolysis for ATP production that is supported by cytosolic reductive metabolism, while preventing this metabolic adaptation can increase the accumulation of ROS and a reduction of metastatic capacity of cancer cells (82). In addition, the metabolic interaction between cancer cell mitochondrial respiration and catabolism in carcinoma-associated fibroblasts enhances the growth and metastasis of cancer cells (83). The expression of miR-485 is downregulated in breast cancer tissues and the overexpression of its both mature forms, miR-485-3p and miR-485-5p, restrains mitochondrial respiration and suppresses the metastasis of breast cancer cells by downregulating peroxisome proliferator-activated receptor-gamma coactivator (PGC)−1α (84).

It is well known that OXPHOS produces more ATP than glycolysis under the same condition (13). Studies used to suggest that OXPHOS plays a minor role in the energy metabolism of tumor cells due to their deficient mitochondrial functions. However, a study shows that 80% of the energy generated by MCF-7 breast cancer cells *in vitro* is derived from mitochondrial OXPHOS and only 20% from glycolysis (25). Another study also finds that genes involved in OXPHOS are significantly upregulated in breast, leukemia, lung, lymphoma and ovarian cancers (85). Therefore, tumor cells may use aerobic glycolysis for energy and glycolytic metabolites are transferred into anabolic pathways to support malignancy, but this process may be suspended during cancer cell metastasis. In support, it is shown that aggressive cancer cells favor OXPHOS for energy supply (86). Although the dispute exists, it seems that OXPHOS is also an important metabolic pattern of tumor cells (87).

It is unclear how primary and metastatic tumors select different metabolic pathways, glycolysis or OXPHOS, for their energy supply. However, it is acceptable that glycolysis is more remarkable in aggressive and fast-growing tumors, and different types of cancer may have different metabolism pathways. A subset of glioma cells relies on glycolysis while the others in the same tumor depend on OXPHOS for energy supply, indicating a characteristic of metabolic heterogeneity (88). A genetic analysis shows that breast cancer cells utilizing glycolysis as the main energy metabolic method prefer to metastasize to the liver, but cells adopting OXPHOS as the principal metabolic pathway are more likely to metastasize to bone and lungs, and pyruvate dehydrogenase kinase-1 (PDK1) is required for liver metastasis (89). Despite metabolic heterogeneity that exists in the same tumor and metabolic types affect the organs where tumor cells metastasize, little information is available regarding the influence of ncRNAs on OXPHOS. A study suggests that miR-155 and miR-210 derived from melanoma exosomes promote glycolysis and inhibit OXPHOS in tumor cells and contribute to the creation of a pre-metastatic niche (90).

### Lipid Metabolism

Lipid anabolism is an important indicator of abnormal tumor metabolism because lipids provide components of biofilms and regulate fluidity and lipid molecule signal transduction of cytomembranes, and thus participates in the metastasis of cancer cells (91, 92). Under metabolic stress, tumor cells strengthen the coupling of fatty acid anabolism and catabolism to establish a fatty acid circulation network to promote their growth and metastasis (93).

In a mouse model with subcutaneous osteosarcoma, the serum metabolic profiling reveals an increase of key metabolites in glycolysis and tricarboxylic acid cycle (TCA); while in mice with lung metastasis, serum metabolic profile shows a decrease of most metabolites, except for cholesterol and free fatty acids, suggesting that elevated lipid metabolism may be associated with tumor metastasis (94). Sterol regulatory element-binding transcription protein 1 (SREBP1), a candidate target of miR-18a-5p, is the master transcription factor that controls lipid metabolism; and miR-18a-5p can suppress the invasion and migration of breast cancer cells by regulating SREBP1 (48). MiR-661 is required for the efficient invasion of breast cancer cells by destabilizing StAR-related lipid transfer protein 10 and the cell-cell adhesion protein Nectin-1, leading to the downregulation of epithelial markers (49). Acetyl-CoA carboxylase (ACC), fatty acid synthase (FASN) and 3-hydroxy-3-methyl-glutaryl-CoA reductase (HMGR) are potential targets of miR-195, whose ectopic expression regulates cellular triglyceride and cholesterol levels, leading to decreased proliferation and metastasis of breast cancer cells (50). Hypoxia decreases the expression of miR-409-3p, a regulator of FABP4 (fatty acid binding protein 4), which increases the metastatic potential of ovarian cancer cells (51).

Fatty acid anabolism requires the activation of ACC, adenosine triphosphate citrate lyase (ACLY) and FASN (93), which are upregulated in aggressive tumors and associated with a poor prognosis (95, 96). ACLY participates in the *de novo* synthesis of fatty acids by converting citrate into oxaloacetate and cytosolic acetyl-CoA. In gastric cancer, highly expressed ACLY is closely related to advanced stages and lymph metastasis (97). ACLY regulates low molecular weight isoform of cyclin E (LMW-E), which can also enhance the enzyme activity of ACLY in a positive feedback way, promoting the formation of lipid droplets and the metastasis of breast cancer cells (98). ACLY stabilizes CTNNB1 (beta-catenin 1) protein and enhances its transcriptional activity, thus promoting the migration and invasion of colon cancer cells (99). MiR-22 restricts the metastasis of breast cancer cells by inhibiting the expression of ACLY (52). Overexpression of miR-133b in gastric cancer increases the levels of nuclear PPAR-γ, which decreases the transcriptional activity of ACLY, and then represses the invasion of cells (53). ACC is a rate-limiting enzyme for fatty acid synthesis, which catalyzes acetyl-CoA to form malonyl-CoA (100). ACC1 is highly modulated by phosphorylation and allosteric regulation and plays a key role in promoting a speedy adaptation to novel microenvironments (101). In HCC, highly expressed ACC1 is closely related to poor differentiation, vascular invasion, and poor prognosis, and has been regarded as a biomarker for early diagnosis (102). Contradictorily, ACC1 inhibition is shown to promote the metastasis of breast cancer cells (103), indicating the roles of ACC1 may be cancer type-dependent. Acyl-CoA oxidase 1 (ACOX1) is a key enzyme of the fatty acid oxidation pathway and its overexpression alleviates the migration and invasion of colorectal cancer (104). The class III deacetylase sirtuin 1 (SIRT1) prevents the trans-activation effect of activator protein (AP-1) on miR-15b-5p by deacetylation of AP-1, then upregulates the expression of ACOX1, which act as a direct target for miR-15b-5p (104).

Fatty acid catabolism also plays a role in the process of cancer metastasis. Monoacylglycerol lipase, functioning with hormone-sensitive lipase (LIPE) to hydrolyze intracellular triglyceride stores to fatty acids and glycerol, is highly expressed in prostate cancer and is related to the EMT process (105). Phospholipase D (PLD), an enzyme that hydrolyzes phosphatidylcholine to produce the signal molecule phosphatidic acid and soluble choline, promotes the metastasis of cancer cells (106). Adipocytes are the carrier of energy, sources of hormones and cytokines, and a crucial component of tumor microenvironments, and also facilitate tumor metastasis. For instance, melanoma cells have a higher level of fatty acid oxidation after they absorb fatty acid oxidase enzymes in exosomes secreted by adipose cells (107). However, the roles of ncRNAs in fatty acid catabolism and adipocytes remain to be clarified.

### Glutamine Metabolism

Cancer cells have shown increased glutamine uptake and catabolism. Glutaminase (GLS) catalyzes glutamine into glutamate, which is subsequently catalyzed by glutamate dehydrogenase (GDH) to form α-ketoglutarate, finally entering the TCA as an important energy source. Although glutamine is a non-essential amino acid, it is indispensable in specific conditions and its metabolism has important biological significance for cancer cells. Glutamine can increase the expression of hypoxia-inducible factor (HIF)-1α, enhance the pro-autophagic effect of its target BNIP3 (BCL2/adenovirus E1B 19kDa interacting protein 3), and promote the metastasis of melanoma cells (108). Metabolite fumarate, an intermediate product of glutamine metabolism, reduces the level of ROS and maintains the balance of redox by activating glutathione peroxidase, and thus promoting the metastasis of cancer cells (109). C-Myc is an oncogenic transcription factor and promotes glutamine catabolism to fuel the growth of cancer cells by upregulating GLS and suppresses proline oxidase primarily through upregulating miR-23b (56). LncRNA GLS-AS regulates a feedback loop of glutaminase and c-Myc, thus being involved in the metastasis and representing a therapeutic target for the metabolic reprogramming of pancreatic cancer cells (10). MicroRNA-133a-3p targets GABARAPL1 (gamma-aminobutyric acid receptor-associated protein-like 1) to inhibit autophagy-mediated glutaminolysis, thereby inhibiting metastasis of gastric cancer (110).

## ncRNAs and Metabolic Signaling Pathways

**Figure 3:**
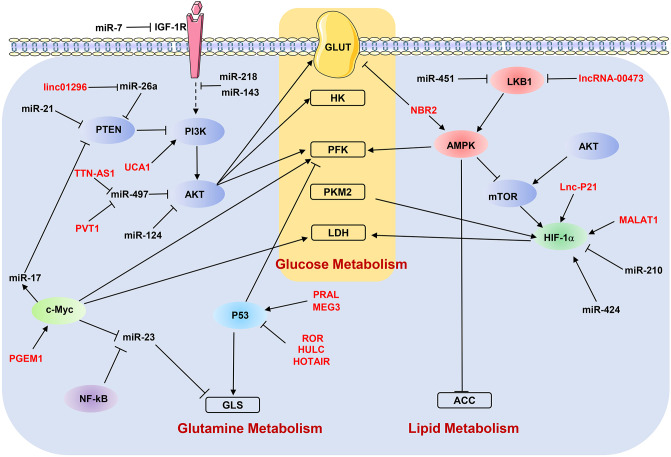
NcRNAs regulate metabolic reprogramming by targeting signaling pathways. “→” indicates positive regulation or activation; “⊥”, negative regulation or blockade; dotted lines “—–”, indirect regulation. LncRNAs are marked as red color. ACC, acetyl-CoA carboxylase; AMPK, AMP-activated protein kinase; GLS, glutaminase; GLUT, glucose transporter; HK, hexokinase; IGF-1R, insulin-like growth factor-1 receptor; LDH, lactate dehydrogenase; LKB1, liver kinase B1; PFK, phosphofructokinase; PKM2, pyruvate kinase M2; PTEN, phosphatase and tensin homolog.

As discussed above, ncRNAs participate in the metastasis-related metabolic reprogramming by regulating individual genes. In the following paragraphs, we discuss how dysregulated ncRNAs modulate the key metabolic signaling pathways to affect the metastasis of cancer cells (Figure 3).

### Hypoxia-Inducible Pathways

Hypoxic microenvironments are very frequently observed in almost all the solid tumors, have an impact on cell biological behaviors and extracellular matrix remodeling, increase metastatic capacities and contribute greatly to therapy resistance (111). Under aerobic conditions, tumor cells have a high rate of glucose-dependent metabolism (112), while in hypoxic microenvironments they have to adjust themselves to lower oxygen situations by changing the metabolic pattern, resulting in the suppression of anti-cancer immunity and high potential capacities of invasion and migration (113). HIFs are the master driving forces of the cellular adaption to hypoxia and well-known transcription factors by regulating a vast array of genes involved in angiogenesis and metastasis, and in particular, GLUTs and glycolytic enzymes including HK2, 6-phosphofructokinase, and LDHA are regulated by HIFs (111). The expression and stabilization of HIFs are controlled by mRNAs. For example, miR-365 directly targets homeobox A9 (HOXA9) by binding to 3′-UTR region, and the downregulation of HOXA9 increases the expression of HIF-1α and its downstream glycolytic genes HK2, GLUT1, and PDK1, promoting glycolysis and metastasis of cutaneous squamous cell carcinoma (114). The knockdown of miR-592 in HCC cells strengthens glycolysis by enhancing WSB1-induced HIF-1α stability and promotes HCC cell migration *in vitro* (115).

### LKB1-AMP Activated Protein Kinase Pathway

AMPK (5′ adenosine monophosphate-activated protein kinase) is a critical sensor to maintain cellular energy homeostasis. AMPK phosphorylates ACC1 and SREBP1 to inhibit the synthesis of fatty acids, cholesterol, and triglycerides, and activate fatty acid uptake. It also stimulates glycolysis by activating phosphorylation of PFKFB3 and glycogen phosphorylase (116). Downregulating AMPK shows a promoting effect on the growth and biosynthesis of cancer cells (117), and many types of cancer cells have a shortage of AMPK to maintain their glycolytic phenotypes (16). Activating AMPK is required for an increased AMP/ATP ratio and switching the oxidative metabolic to glycolytic phenotypes (116). Liver kinase B1 (LKB1) is a tumor suppressor that locates at the upstream of AMPK and can repress ATP depletion by phosphorylating and activating AMPK when cellular ATP levels are limited (118). Under tumor microenvironments, miR-7 inhibits autophagy by upregulating the LKB1-AMPK signaling pathway, leading to a reduced intracellular glucose supply, and as a result, the proliferation and metastasis of pancreatic cancer cells are inhibited (119). MiR-451 regulates the proliferation, migration and responsiveness to glucose deprivation of glioma cells by targeting the LKB1/AMPK pathway, and depresses the LKB-1-associated protein CAB39 that promotes glioma cells adapting to metabolic stress (120). Taurine up-regulated gene 1 (TUG1) is highly expressed in HCC cells and upregulates miR-455-3p at the transcriptional level, thus the TUG1/miR-455-3p/AMPKβ2 axis promotes glycolysis and metastasis by upregulating HK2 (40). Higher expression of lncRNA MACC1-AS1 correlates with the lung metastasis of gastric cancer cells, and MACC1-AS1 is elevated under metabolic stress and facilitates metabolic plasticity by increasing MACC1 mRNA expression and strengthening glycolysis and anti-oxidative abilities via the AMPK/Lin28 pathway (121). LncRNA MACC1-AS1 is also upregulated in pancreatic cancer and related to poor prognosis, and its knockdown inhibits the metastasis of pancreatic cancer cells by upregulating the expression of PAX8 (paired-box gene 8), which plays a role in activating NOTCH1 signaling and promoting cell aerobic glycolysis (122).

### PI3K/AKT/mTOR Pathway

In tumor cells, the classical PI3K/AKT/mTOR signaling pathway is often highly expressed and is involved in regulating cancer growth, proliferation, invasion, and metastasis (123, 124). Recent studies also show that PI3K/AKT/mTOR pathway also functions in tumor cell metabolisms such as glycolysis (125), lipid metabolism (126) and amino acid metabolism (127). Meanwhile, ncRNAs also participate in the regulation of cell metabolism through the PI3K/AKT/mTOR pathway, thus affecting tumor cell metastasis. MiR-204-5p expressed in breast cancer cells affects the mTOR pathway, reduces the oxygen consumption and extracellular acidification rates, and inhibits metastasis (128). Circular RNA circNRIP1 acts as ceRNA to bind to miR-149-5p, activates the AKT/mTOR signaling pathway, thus increases glucose uptake, lactate contents and ATP production, regulates the Warburg effect and promotes gastric cancer cell metastasis (129). LINC00963 promotes non-small cell lung cancer metastasis by preventing ubiquitination of glycolytic kinase PGK1 (phosphoglycerate kinase 1), which activates the AKT/mTOR pathway (130). MiR-384 regulates the expression of pleiotrophin, which can upregulate lipogenic genes, mediate *de novo* lipid synthesis and promote the metastasis of HCC cells via the N-syndecan/PI3K/Akt/mTOR pathway (131).

## Conclusion and Expectation

**Figure 4:**
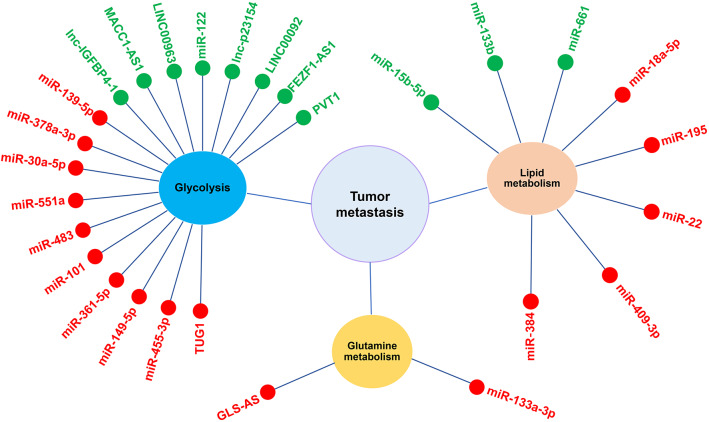
NcRNAs participate in tumor metastasis by regulating metabolic reprogramming. Red and green pellets represent ncRNAs that inhibit and promote, respectively, the metastasis through regulating glycolysis, lipid metabolism or glutamine metabolism.

Metastasis is a major obstacle for successful treatments of cancer and represents the leading cause of cancer-related death. Metabolic reprogramming is another hallmark of cancer because cancer cells obtain their energy supply mainly depending on glycolysis rather than mitochondrial oxidative phosphorylation through altered oncogenic metabolic pathways. The changes in metabolisms of glucose, lipid, and glutamine and mitochondrial respiration and oxidative phosphorylation involve the hypoxia-inducible, LKB1-AMP activated protein kinase and other signaling pathways in cancer cells. These metabolic alterations render cancer cells obtaining energy and metabolites for fast bioenergetics and metabolic fluxes, and being prone to metastasize to distant organs, where normal cells also undergo metabolic changes to form pre-metastatic niches for the implantation and growth of cancer cells. Many ncRNAs, particularly miRNAs and lncRNAs, have been identified as major participants in the metabolic gene regulatory networks and the multi-staged process of metastasis. Some of them either inhibit or promote cancer metastasis involving cell motility, transit in the circulation, and growth at a new site by regulating the metabolism of glycolysis, lipid, and glutamine (Figure 4).

Despite cumulative studies showing the altered expression profiles of ncRNAs during metabolic rearrangement in cancer, their roles and molecular characteristics remain largely unexplored. Without a doubt, the dysregulation of ncRNAs influences multiple metabolic processes and plays a critical role in tumor metastasis. More ncRNAs are being identified as potential diagnostic biomarkers or therapeutic targets for cancers. For example, a meta-analysis of a large cohort of cancer patients reveals that lncRNA SNHG12 serves as a diagnostic and prognostic biomarker and a druggable therapeutic target with promising clinical potential in multiple types of cancer (132). MiR-34, miR-16, and miR-155 have been regarded as cancer therapeutic targets and are being evaluated in clinical trials (133). Furthermore, therapeutic small RNA drugs are becoming novel promising therapeutics since the first small-interfering RNA (siRNA) drug, Patisiran, which acts by binding and degrading transthyretin mRNA, was approved for the treatment of a rare polyneuropathy by FDA in 2018. However, it seems that ncRNAs are likely to play “fine-tuning” rather than “definite” roles. NcRNAs interact with each other and with other types of factors to form complicated networks, which regulate metabolic reprogramming and metastasis of cancer cells. Therefore, a deeper and more comprehensive understanding of the complicated networks of interactions that ncRNAs coordinate in the metabolic and metastatic processes may help translate the discoveries into a strategy for the diagnosis and treatment of cancer (32).

In addition, thousands of ncRNA sequences exist within cells and more types of ncRNAs have been recently discovered, such as circular RNA (circRNA), piwi-interacting RNA (piRNA), small interfering RNAs (siRNA), enhancer RNAs (eRNA) and promoter-associated RNA (PAR), which engage in cellular processes including chromatin remodeling, transcription, post-transcriptional modifications and signal transduction (134, 135). Further investigation on the new ncRNAs and their interactions with other ncRNAs and the associated networks may provide a unique opportunity to elucidate underlying mechanisms of how ncRNAs operate in the crosstalk between cancer metabolic reprogramming and metastasis.

## Author Contributions

XS designed the study and finalized the manuscript. ZL drafted the manuscript. Both authors approved the final manuscript.

## Conflict of Interest

The authors declare that the research was conducted in the absence of any commercial or financial relationships that could be construed as a potential conflict of interest.

## Abbreviations

ACC: acetyl-CoA carboxylase
ACLY: adenosine triphosphate citrate lyase
ACOX1: Acyl-CoA oxidase 1
AMPK: AMP-activated protein kinase
AMPKβ2: adenosine monophosphate-activated protein kinase subunit β2
ATP: adenosine triphosphate
BNIP3: BCL2/adenovirus E1B 19kDa interacting protein 3
ceRNA: competing endogenous RNA
CKB: creatine kinase brain-type
CTNNB1: beta-catenin 1
CRC: colorectal cancer
EMT: epithelial-mesenchymal transition
FABP4: fatty acid binding protein 4
FASN: fatty acid synthase
FBXL3: F-box/LRR-repeat protein 3
FDA: Food and Drug Administration
PFKFB2: 6-phosphofructo-2-kinase/fructose-2,6-biphosphatase 2
FGFR1: fibroblast growth factor receptor 1
GABARAPL1: gamma-aminobutyric acid receptor-associated protein-like 1
GBC: gallbladder cancer
GDH: glutamate dehydrogenase
GLS: glutaminase
GLUT: glucose transporter
HCC: hepatocellular carcinoma
HIF: hypoxia-inducible factor
HK: hexokinases
HMGR: 3-hydroxy-3-methyl-glutaryl-CoA reductase
H_2_O_2_: hydrogen peroxide
HOXA9: Homeobox A9
siRNA: small-interfering RNA
LDH: lactate dehydrogenase
LKB1: liver kinase B1
LIPE: hormone-sensitive lipase
LMW-E: low molecular weight isoform of cyclin E
lncRNA: long-chain non-coding RNA
MMP: metalloproteinase
miRNA: microRNA
ncRNA: non-coding RNA
NOTCH1: Notch homolog 1
OGT: O-GlcNAcylation transferase
OXPHOS: oxidative phosphorylation
PAX8: paired-box gene 8
PDK1: pyruvate dehydrogenase kinase 1
PFKFB: 6-phosphofructo-2-kinase/fructose-2,6-biphosphatase
PGC: peroxisome proliferator-activated receptor gamma coactivator
PGK1: phosphoglycerate kinase 1
PLD: Phospholipase D
PKM2: pyruvate kinase M2
PPAR: peroxisome proliferator activated receptor
ROS: oxygen species
SREBP1: sterol regulatory element binding transcription protein 1
TCA: tricarboxylic acid cycle
TGF-β: transforming growth factor-β
TGIF2: TGF-β-induced factor homeobox 2
TME: tumor micro-environment
UDP-GlcNAc: UDP-β-DN-acetylglucosamine
3′-UTR: three prime untranslated region.
